# BMC Veterinary Research reviewer acknowledgement 2014

**DOI:** 10.1186/s12917-015-0320-1

**Published:** 2015-02-19

**Authors:** Hayley Henderson

**Affiliations:** BioMed Central, Floor 6, 236 Gray’s Inn Road, London, WC1X 8HB UK

## Contributing reviewers

**Leticia Abecia**

Spain

**Gerardo Acosta**

Chile

**Pier Luigi Acutis**

Italy

**Alexandra Adams**

UK

**Hippolyte Affognon**

Kenya

**Claudio Afonso**

USA

**Jose Aguilera**

Spain

**Hiroyuki Akagi**

Japan

**Pablo Alarcon**

UK

**Samer Alasaad**

Spain

**Séverine Albouy-Boyer**

Canada

**Noelia Aldai**

Spain

**Karin Allenspach**

UK

**Julio Alvarez**

Spain

**Gema Alvarez-García**

Spain

**Carlos Ambrosio**

Brazil

**Burim Ametaj**

Canada

**Hermann Ammer**

Germany

**Janetrix Hellen Amuguni**

USA

**Arturo Anadon**

Spain

**Kevin Anderson**

USA

**Magnus Carl Andersson**

Finland

**Gunther Antonissen**

Belgium

**Luis Antunes**

Portugal

**Haseeb Anwar**

Pakistan

**Michal Arabski**

Poland

**Virginia Aragon**

Spain

**Toshiro Arai**

Japan

**Debra Archer**

UK

**David Argyle**

UK

**Itamar Aroch**

Israel

**Margarete Arras**

Switzerland

**Karin Artursson**

Sweden

**Chris Ashwell**

USA

**Spiridoula Athanasiadou**

UK

**Graeme Attwood**

New Zealand

**Joerg Auer**

Switzerland

**Gaël Auray**

Switzerland

**Mian Muhammad Awais**

Pakistan

**Chrisostom Ayebazibwe**

Uganda

**Ignacio Badiola**

Spain

**Kris Baert**

Belgium

**Wolfgang Baeumer**

USA

**Emilia Bagnicka**

Poland

**Tamás Bakonyi**

Hungary

**Vinayagamurthy Balamurugan**

India

**Udeni Balasuriya**

USA

**Alfonso Baldi**

Italy

**Cynthia Baldwin**

USA

**Anne Balkema-Buschmann**

Germany

**Hywel Ball**

UK

**Ray Ball**

USA

**Jacek Bania**

Poland

**Claudio Barbeito**

Argentina

**Lisa Barco**

Italy

**Alistair Barr**

UK

**Maria Barrandeguy**

Argentina

**Animesh Barua**

USA

**Giuseppe Baselli**

Italy

**Anna Bassols**

Spain

**Peter Bates**

UK

**John Bauer**

USA

**Maximilian Baumann**

Germany

**Gregoy Bedecarrats**

Canada

**Aynun Begum**

USA

**Elsa Beltran**

UK

**Livia Benato**

UK

**David Bennett**

UK

**Olaf Berke**

Canada

**Dirk Berkvens**

Belgium

**Emma Bermingham**

New Zealand

**Paul Bessell**

UK

**V Bhanuprakash**

India

**Tomasz Bielecki**

Poland

**Barbara Biller**

USA

**Sarah Billeter**

USA

**John Bingham**

Australia

**Helen Birch**

UK

**Karyn Bischoff**

USA

**Francois Blachier**

France

**Pat Blackall**

Australia

**Barbara Blacklaws**

UK

**Damer Blake**

UK

**Sandra Blome**

Germany

**Gerd Bobe**

USA

**Barbara Bockstahler**

Austria

**Michael Böer**

Germany

**Patrick Boerlin**

Canada

**Josef Böhm**

Austria

**Anders Miki Bojesen**

Denmark

**J. Elizabeth Bolhuis**

Netherlands

**Diego Bonatto**

Brazil

**Alexandro Bonifaz**

Mexico

**Stephanie Booth**

Canada

**Manuel Vitor Borca**

USA

**Nicole Borel**

Switzerland

**Susanne Boroffka**

Netherlands

**Luca Borrelli**

Italy

**Uberto Bortolotti**

Italy

**Tim Bosmans**

Belgium

**Gianluca Bossi**

Italy

**Adrian Boswood**

UK

**Filip Boyen**

Belgium

**Barry Bradford**

USA

**Daniel Bradway**

USA

**Gudrun Brandes**

Germany

**Marina Brash**

Canada

**Jackie Brearley**

UK

**Andrew Breed**

UK

**Walter Brehm**

Germany

**Klaus Brehm**

Germany

**Julia Breuer**

Germany

**Gert Breur**

USA

**Robert Briggs**

USA

**Victor Brugman**

UK

**Sebastien Buczinski**

Canada

**Hubert Buczkowski**

UK

**Steven Budsberg**

USA

**Rikke Buhl**

Denmark

**Franciszek Burdan**

Poland

**Laura Burgin**

UK

**Eric Burrough**

USA

**Neil Burton**

UK

**Patrick Butaye**

Belgium

**L Catalina Cabrera**

USA

**Silvia Cabrera**

Spain

**Petra Cagnardi**

Italy

**Jianping Cai**

China

**Hugh Cai**

Canada

**Luigi Calamari**

Italy

**Paolo Calistri**

Italy

**Luis Calvinho**

Argentina

**Begoña Calvo**

Spain

**Zongxi Cao**

China

**Vittorio Capello**

Italy

**Tereza Cardoso**

Brazil

**Jorge U. Carmona**

Colombia

**Noèlia Carrasco**

Spain

**Angel L. Carrascosa**

Spain

**Jose M Carrillo Poveda**

Spain

**Jorge Castro**

Spain

**Elena Catelli**

Italy

**Lissandra Cavalli**

Brazil

**Vladimir Celer**

Czech Republic

**Jose Ceron**

Spain

**Giuliano Cerulli**

Italy

**Chanhee Chae**

Korea, South

**Chia-Yi Chang**

Taiwan

**Shih-Chieh Chang**

Taiwan

**Guillaume Chanoit**

UK

**Aspinas Chapwanya**

Ireland

**Carole Charlier**

Belgium

**Johannes Charlier**

Belgium

**Hso-Chi Chaung**

Taiwan

**Huanchun Chen**

China

**Hong Chen**

China

**Yaosheng Chen**

China

**Anik Chevrier**

Canada

**Adeline Chia**

Malaysia

**Mario Chiari**

Italy

**Ming-Tang Chiou**

Taiwan

**Tomasz Chmielewski**

Poland

**Bhudipa Choudhury**

UK

**Francesco Cian**

UK

**Louise Clark**

UK

**Sarah Cleaveland**

UK

**Archie Clements**

Australia

**Alison Clode**

USA

**Joan Coates**

USA

**Hans Coetzee**

USA

**Gerald Coles**

UK

**Philippe Colson**

France

**Gavin Conant**

USA

**Anthony Confer**

USA

**Franz Josef Conraths**

Germany

**Ronald Corbee**

Netherlands

**Guillaume Counotte**

Netherlands

**Wendel Coura-Vital**

Brazil

**Christopher Cowled**

Australia

**Vasile Cozma**

Romania

**Carolyn Cray**

USA

**Pascale Crepieux**

France

**Juan Cuervo-Arango**

Spain

**William Culp**

USA

**Roman Dabrowski**

Poland

**Trachsel Dagmar**

France

**Benjamin Dainat**

Switzerland

**Stefano D'Amelio**

Italy

**Sven Dänicke**

Germany

**Andrew Davidson**

UK

**Peter Davies**

USA

**Andrew Davison**

UK

**Lucy Davison**

UK

**Astrid De Greeff**

Netherlands

**Ario De Marco**

Slovenia

**Stefaan De Smet**

Belgium

**Stephanie De Vleeschauwer**

Belgium

**Clazien J. De Vos**

Netherlands

**Theo De Waal**

Ireland

**Nicola Decaro**

Italy

**Scott Dee**

USA

**Odir Dellagostin**

Brazil

**Mauro Delogu**

Italy

**Sagi Denenberg**

Canada

**Matthew Denwood**

UK

**Klaus Depner**

Germany

**Veronique Dermauw**

Belgium

**Robert Desrosiers**

Canada

**Johann Detilleux**

Belgium

**Andres Diaz**

USA

**Fco. Javier Diéguez**

Spain

**Chan Ding**

China

**Linda Dixon**

UK

**Cinzia Domeneghini**

Italy

**Mariano Domingo**

Spain

**Monique Dore**

Canada

**Jos Dortmans**

Netherlands

**Sara Downs**

UK

**Barbara Drews**

Switzerland

**Bert Driessen**

Belgium

**Tod Drost**

USA

**Ana Duarte**

Portugal

**Caroline Dube**

Canada

**Damien Dubois**

France

**Edward Dubovi**

USA

**Jennifer Duncan**

UK

**Stephen Dunham**

UK

**Magdalena Dunowska**

New Zealand

**Céline Dupuy**

France

**Michael Dykstra**

USA

**Sue Dyson**

UK

**Francis Dziva**

Trinidad and Tobago

**Peter David Eckersall**

UK

**Syed Ehtisham Ul Haque**

Pakistan

**Yvonne Eiby**

Australia

**Armin Elbers**

Netherlands

**Yvonne Elce**

Canada

**Steve Elder**

USA

**William Ellis**

UK

**Mansour El-Matbouli**

Austria

**Ingegerd Elvers**

USA

**Carsten Enevoldsen**

Denmark

**Francis Enjalbert**

France

**Luis Enjuanes**

Spain

**Kira Epstein**

USA

**Ronald Erskine**

USA

**Mark Erwin**

Canada

**Matthieu Eveillard**

France

**Darrick Evensen**

USA

**Christelle Fablet**

France

**Maria Fahie**

USA

**Mohamed Fakhr**

USA

**Kiterie Faller**

UK

**Yunpeng Fan**

China

**Mark Farnworth**

New Zealand

**Vahab Farzan**

Canada

**Maren Fedrowitz**

Germany

**Daniel Feeney**

USA

**Li Feng**

China

**Aude Ferran**

France

**Edmilson Ferreira De Oliveira Filho**

Belgium

**Carrie Finno**

USA

**Nahuel Fittipaldi**

Canada

**Paul Flecknell**

UK

**Krzysztof Flisikowski**

Germany

**Michael Flythe**

USA

**Steven Foley**

USA

**Anthony Fooks**

UK

**Ivo Foppa**

USA

**Hubert Forster**

Jordan

**Geoffrey Foster**

UK

**Gilles Foucras**

France

**Guillaume Fournié**

UK

**Leo Foyle**

Australia

**Carlos Franco**

Spain

**Dimitrios Frangoulidis**

Germany

**Conrad Freuling**

Germany

**Schirin Friederichs**

Germany

**Anika Friese**

Germany

**Jean-Pierre Frossard**

UK

**George Fthenakis**

Greece

**Clement Furlong**

USA

**Malgorzata Gajewska**

Poland

**Vladimir Galindo-Zamora**

Colombia

**Marina Gallo Calderon**

Argentina

**Patricia Gama**

Brazil

**Gualtiero Gandini**

Italy

**Ana L. Garcia-Perez**

Spain

**Mutien-Marie Garigliany**

Belgium

**David Gasper**

USA

**Charles Gauci**

Australia

**Anne Geffré**

France

**Claudio Genchi**

Italy

**Solange Gennari**

Brazil

**Arcangelo Gentile**

Italy

**Ivaylo Gentschev**

USA

**Raymond Geor**

USA

**Kerstin Gerlach**

Germany

**Pierre Germon**

France

**Andrea Giacometti**

Italy

**Ilias Giannenas**

Greece

**Mirutse Giday**

Ethiopia

**Pedro J. Ginel**

Spain

**Mario Giorgi**

Italy

**Etienne Giraud**

France

**Liz Glass**

UK

**Mindy Glass**

USA

**Helen Golder**

Australia

**Rita Goncalves**

UK

**Lorenzo Gonzalez**

UK

**David González-Barrio**

Spain

**Daniela Gorgas**

Switzerland

**Marga Goris**

Netherlands

**Eamonn Gormley**

Ireland

**Simon Paul Graham**

UK

**Simon Graham**

UK

**Iain Grant**

UK

**Kathryn Graves**

USA

**Gilbert Greub**

Switzerland

**Deborah Grosenbaugh**

USA

**Sigrid Grulke**

Belgium

**Alonso Guedes**

USA

**Roberto Guedes**

Brazil

**Bruce Gummow**

Australia

**Sanjeev Gupta**

Ireland

**Franco Guscetti**

Switzerland

**Carlton Gyles**

Canada

**Adriana Györke**

Romania

**Bernd Haas**

Germany

**Daniela Christina Hadorn**

Switzerland

**Olga Haenen**

etherlands

**Ferry Hagen**

Netherlands

**Mitika Hagiwara**

Brazil

**Micah Hahn**

USA

**Rodrigo Hamede**

Australia

**David Hampson**

Australia

**Jarrad Hampton-Marcell**

USA

**Sanni Hansen**

Denmark

**Tom Harcourt-Brown**

UK

**Timm Harder**

Germany

**Robert Harman**

USA

**Balazs Harrach**

Hungary

**Chris Harris**

Canada

**Kenton Hart**

UK

**Kirsten Hattermann**

Germany

**Eleanor Hawkins**

USA

**Qing He**

USA

**Patricia Hedenqvist**

Sweden

**Joanna Hedley**

UK

**Ibrahim Hegab**

Korea, South

**Nagendra Hegde**

India

**Mohammad Heidari**

USA

**Markus M. Heimesaat**

Germany

**Wayne Hein**

Australia

**Marcos Heinemann**

Brazil

**Lucie Heinzerling**

Germany

**Chris Helps**

UK

**Joerg Henning**

Australia

**Christina Herold**

Germany

**Pedro Herraez**

Spain

**Georg Herrler**

Germany

**Wolfgang Heuwieser**

Germany

**Marion Hewicker-Trautwein**

Germany

**Janet Hill**

Canada

**Gen Hiyama**

Japan

**Douglas Hodgins**

Canada

**Jane Hodgkinson**

UK

**Chris Hodgson**

UK

**Doris Hoeltig**

Germany

**Detlef Hoereth-Boentgen**

Germany

**Martin Hofmann**

Switzerland

**Erik Hofmeister**

USA

**Astrid Hogenkamp**

Netherlands

**Odd Viking Höglund**

Sweden

**Ole Hojberg**

Denmark

**Mark Holmes**

UK

**Shannon Holmes**

USA

**Walther Honscha**

Germany

**Jayne Hope**

UK

**Robert Horton**

UK

**Margaret Hosie**

UK

**Herve Hoste**

France

**Xiuguo Hua**

China

**Bing Huang**

China

**Velia Hülsmeyer**

Germany

**Nora Hunter**

UK

**Jamal Hussaini**

Malaysia

**Changbaig Hyun**

Korea, South

**Eleonora Iacono**

Italy

**Yamamoto Ichiro**

Japan

**Jean-Claude Ionita**

Germany

**Re Isaacson**

USA

**Gloria Isani**

Italy

**Ramiro Isaza**

USA

**Naoki Isobe**

Japan

**Daisuke Ito**

USA

**Abdul Jabbar**

Australia

**Katarzyna Jagusztyn-Krynicka**

Poland

**George Jallo**

USA

**Henrik Elvang Jensen**

Denmark

**Trine Jensen**

Denmark

**Marianne Jensen-Waern**

Sweden

**Narayanaperumal Jeyathilakan**

India

**Jieyuan Jiang**

China

**Yonghou Jiang**

China

**Amy Johnson**

USA

**Christopher Johnson**

USA

**Timothy Johnson**

USA

**Clinton Jones**

USA

**Richard Jones**

UK

**Ronald Jones**

UK

**Chaitanya Joshi**

India

**Jennifer Lee Juengel**

New Zealand

**Jana Juránková**

Czech Republic

**Ramon A. Juste**

Spain

**Jaroslaw Kaba**

Poland

**Annemarie Kaesbohrer**

Germany

**Donata Kalthoff**

Germany

**Joshua Kamani**

Nigeria

**Ji-Houn Kang**

Korea, South

**Heike Kaspar**

Germany

**Anil Kumar Kataria**

India

**Frank Katzer**

UK

**Kevin Keegan**

USA

**Rob Kelly**

UK

**Michael Kent**

USA

**Mahesh Khatri**

USA

**Kamel Khazal**

USA

**Timothy Kidd**

Australia

**Manfred Kietzmann**

Germany

**Amy Kilbride**

UK

**Hyeun Bum Kim**

Korea, South

**Ralph Kirby**

Taiwan

**Carsten Kirkeby**

Denmark

**Jolle Kirpensteijn**

Netherlands

**William Kisseberth**

USA

**Jerzy Kita**

Poland

**Anna-Mariam Kiviranta**

Finland

**Wolfgang Klee**

Germany

**Eyal Klement**

Israel

**Robert Klopfleisch**

Germany

**Derek Knottenbelt**

UK

**Guus Koch**

Netherlands

**Achim Kohler**

Norway

**Nicholas Komar**

USA

**Ganesh Kondabattula**

India

**Timm Konold**

UK

**Peter Kook**

Switzerland

**Hendrik-Jan Kranenburg**

Netherlands

**Peter Krawczel**

USA

**Magdalena Krol**

Poland

**Christa Kuehn**

Germany

**Hanna Kulig**

Poland

**Sachin Kumar**

India

**Nina Kung**

Australia

**Jacqueline Kupper**

Switzerland

**Nitin Kurkure**

India

**Ohkyeong Kweon**

Korea, South

**Roberto La Ragione**

UK

**Raphael Labens**

UK

**Jean-Paul Lallès**

France

**Abraham Landa**

Mexico

**Wil J.M. Landman**

Netherlands

**Paul Langford**

UK

**Carlos Lanusse**

Argentina

**Sasha Lanyon**

Australia

**Susanna Lau**

China

**Marcia Laurenti**

Brazil

**Richard Laven**

New Zealand

**Michael Lawman**

Finland

**Patricia Lawman**

USA

**Yves Le Loir**

France

**John Lednicky**

USA

**Jun Heon Lee**

Korea, South

**Tosso Leeb**

Switzerland

**Peter Lees**

UK

**Chu-Zhao Lei**

China

**Tiziana Lembo**

UK

**Carlo Leonetti**

Italy

**Henry L'Eplattenier**

UK

**Michael Leschnik**

Austria

**Bruno Levecke**

Belgium

**Charles Ley**

Sweden

**Jacqueline Ley**

Australia

**Erguang Li**

China

**Meihang Li**

Canada

**Robert Li**

USA

**Qu Liandong**

China

**Ai-Ho Liao**

Taiwan

**Geneviève Libeau**

France

**Brandon Lillie**

Canada

**Hai Lin**

China

**David Lindsay**

USA

**Susan Little**

Canada

**Chao-Lin Liu**

Taiwan

**Huili Liu**

China

**Changming Liu**

China

**Wenjun Liu**

China

**Qinfang Liu**

USA

**Xiufan Liu**

China

**Caryl Lockhart**

Italy

**Hannes Lohi**

Finland

**Sam Long**

Australia

**Alessio Lorusso**

Italy

**Marta Lourenco**

Belgium

**James Lowe**

USA

**Huaguang Lu**

USA

**Yu Lu**

China

**George Lubas**

Italy

**Stephen Luby**

USA

**Cecilia Lucero Estrada**

Argentina

**Jennifer Luff**

USA

**Stelio Luna**

Brazil

**Heidi Sjetne Lund**

Norway

**Xiaohu Luo**

China

**Agnese Lupo**

Switzerland

**Andrea Luppi**

Italy

**Bertrand Lussier**

Canada

**Inna Lysnyansky**

Israel

**N James Maclachlan**

USA

**James Macleod**

USA

**Dominiek Maes**

Belgium

**Raul Mainar-Jaime**

Spain

**Francesca Mancianti**

Italy

**Christoph Mans**

USA

**Karen Mansfield**

UK

**Lucia Manso-Silvan**

France

**Shengyong Mao**

China

**Ricardo Marcos**

Portugal

**Cinzia Marianelli**

Italy

**Lieta Marinelli**

Italy

**Gabriele Marino**

Italy

**Marga Martin**

Spain

**Guy-Pierre Martineau**

France

**Fernando Martinez**

Spain

**Silvia Martinez-Subiela**

Spain

**Daniele Martins**

Brazil

**Gabriel Martins**

Brazil

**Gift Matope**

Zimbabwe

**Augusto Matos**

Portugal

**Olga Matos**

Portugal

**Jelle Matthijnssens**

Belgium

**Meghan May**

USA

**Christie Mayo**

USA

**Brad Mccall**

Australia

**Scott Mcclure**

USA

**Neil Ross Mcewan**

UK

**John Mcgarry**

UK

**Colin Mcinnes**

UK

**Mary-Louise Mclaws**

Australia

**Barbara Mcmahill**

USA

**Bret Mcnabb**

USA

**Steven Mcorist**

UK

**Graham Medley**

UK

**Joanne Meers**

Australia

**Björn Meij**

Netherlands

**Antonio Melendez Lazo**

Spain

**Adriana Méndez Bernal**

Mexico

**Michael Mendl**

UK

**Estelle Méroc**

Belgium

**Alfred Merritt**

USA

**Ignacio Mesa-Sánchez**

Spain

**Andrea Mess**

Brazil

**Francois Meurens**

Canada

**Martin Michaelis**

UK

**Andrei Daniel Mihalca**

Romania

**Djordje Miljkovic**

Serbia

**Patti Miller**

USA

**Suzanne Millman**

USA

**L Scott Mills**

USA

**Rowan Milner**

USA

**Jose Roberto Mineo**

Brazil

**Takuya Mizuno**

Japan

**Shusei Mizushima**

Japan

**David Modrý**

Czech Republic

**Giovanni Mogicato**

France

**Fouad Mohammad**

Iraq

**Matlou Mokgotho**

South Africa

**Jan Mol**

Netherlands

**Fabiano Montiani-Ferreira**

Brazil

**J. Alberto Montoya-Alonso**

Spain

**Simon More**

Ireland

**Maxim Moreau**

Canada

**Anabela Moreira**

Portugal

**Ana Moreno**

Italy

**Juan Morgaz**

Spain

**Nobuko Mori**

Japan

**Luca Motta**

UK

**Vladimir Mrljak**

Croatia

**Gideon D Mshelia**

Nigeria

**Martha Mulks**

USA

**Thomas Muller**

Germany

**Joachim Müller**

Switzerland

**Lina Mur**

Spain

**Atsushi Murai**

Japan

**Pamela Murison**

UK

**Serge Muyldermans**

Belgium

**Hanspeter Naegeli**

Switzerland

**Vinny Naidoo**

South Africa

**Shan Naidu**

USA

**Bindu Nanduri**

USA

**Richard Nap**

Argentina

**Sebastian Napp**

Spain

**Heiko Nathues**

Switzerland

**Helena Neumayerova**

Czech Republic

**Mandy Nevel**

UK

**Tzi Bun Ng**

Hong Kong

**Seongwon Nho**

Korea, South

**Robin Nicholas**

UK

**Tracy Nicholson**

USA

**Artur Niedzwiedz**

Poland

**Søren Achim Nielsen**

Denmark

**Jarkko Niemi**

Finland

**Stijn Niessen**

UK

**Jorge Nieto**

USA

**Akbar Nikkhah**

Iran

**Flobert Njiokou**

Cameroon

**Chiara Noli**

Italy

**Sarah North**

UK

**Erik Noschka**

Australia

**Christopher Ober**

USA

**Peter Thomas Oberem**

South Africa

**Victoria Anne Offord**

UK

**Hiroyuki Okada**

Japan

**Shoshiro Okada**

Japan

**Yuki Okada**

Japan

**Teresa Cristina Oliveira-Sequeira**

Brazil

**Thierry Olivry**

USA

**Pal A. Olsvik**

Norway

**Oluyinka Olukosi**

UK

**Maarten Oosterlinck**

Belgium

**Tanja Opriessnig**

UK

**Geert Opsomer**

Belgium

**Erman Or**

Turkey

**Angel Ortiz-Pelaez**

UK

**Melania Osto**

Switzerland

**Tarja Pääkkönen (Previously Jokinen)**

Finland

**José Ricardo Pachaly**

Brazil

**Akos Pakozdy**

Austria

**Saverio Paltrinieri**

Italy

**Lisa Pang**

UK

**Fernando Paolicchi**

Argentina

**Elias Papadopoulos**

Greece

**Kostas Papasouliotis**

UK

**Roberto Papini**

Italy

**Bart Pardon**

Belgium

**Elizabeth Parker**

Australia

**Tim Parkin**

UK

**Thomas Parsons**

USA

**Peter Pascoe**

USA

**Santiago Pascual**

Spain

**Urszula Paslawska**

Poland

**Josep Pastor**

Spain

**Gavin Paterson**

UK

**Nina Patzke**

South Africa

**Heather Paxton**

UK

**Rita Payan Carreira**

Portugal

**Laura Peachey**

UK

**David Pearl**

Canada

**Dannele Peck**

USA

**Christian Peham**

Austria

**Xin-Hai Pei**

USA

**Simone Peletto**

Italy

**Li Peng**

China

**Bruno Penna**

Brazil

**Louis Penning**

Netherlands

**Mary Louise Penrith**

South Africa

**Felipe Perecin**

Brazil

**Sandro Pereira**

Brazil

**Andres Perez**

USA

**Dolores Perez Alenza**

Spain

**Bernat Pérez De Val**

Spain

**Jose Perez-Casal**

Canada

**Michael Peterson**

USA

**Rob Pettitt**

UK

**Thilo Pfau**

UK

**Dirk Pfeiffer**

UK

**Adrian Philbey**

UK

**Giuseppe Piccione**

Italy

**Alessandra Piccirillo**

Italy

**Kim Picozzi**

UK

**Tina Pihl**

Denmark

**Gina Pinchbeck**

UK

**Eric Pinloche**

UK

**Patrick Pithua**

USA

**Alessandro Poli**

Italy

**Iuliana Popa**

France

**Thibaud Porphyre**

UK

**Heidrun Potschka**

Germany

**Andrew Potts**

South Africa

**Barbara Powers**

USA

**Barbara Pratscher**

Austria

**Katy Proudfoot**

USA

**Chris Proudman**

UK

**Anna Puigdemont**

Spain

**Felisbina Queiroga**

Portugal

**Rupert Quinnell**

UK

**Wagner Quintilio**

Brazil

**Chantal Ragetly**

France

**Sheila Canevese Rahal**

Brazil

**Zia Rahman**

Pakistan

**Pascal Rainard**

France

**Ana S. Ramirez**

Spain

**Alejandro Ramirez**

USA

**Vijay Ramiya**

USA

**Thaha Rasool**

United Arab Emirates

**Nicki Reed**

UK

**Phil Reeves**

Australia

**Ralf Regenthal**

Germany

**Javier Regidor-Cerrillo**

Spain

**Habib Rehman**

Pakistan

**Michael Reichel**

Australia

**Richard Reid-Smith**

Canada

**Janin Reifenrath**

Germany

**Kerstin Reimers**

Germany

**Craig Reinemeyer**

USA

**William Reisen**

USA

**Monika Reissmann**

Germany

**Alexander Reiter**

USA

**Anne Relun**

France

**David Renaudeau**

Finland

**Subramaniam Renuka**

USA

**Crawford Revie**

Canada

**Brice Reynolds**

France

**Federica Riccardo**

Italy

**Rose Eli Rici**

Brazil

**Christopher Riley**

New Zealand

**Gerald Rimbach**

Germany

**Laura Rinaldi**

Italy

**Robert Ringseis**

Germany

**Cecilia Robat**

USA

**Helen Roberts**

UK

**Mara Rocchi**

UK

**Dominique Rocha**

France

**Annie Rodolakis**

France

**Fernando Rodriguez**

Spain

**Carlos Rodriguez**

USA

**Simon Roe**

USA

**Bernard Roelen**

Netherlands

**Cecilia Rohdin**

Sweden

**Angela Römer-Oberdörfer**

Germany

**Paola Roncada**

Italy

**Ruben Rosales**

UK

**Ricardo Rosenbusch**

USA

**Alexander William Ross**

UK

**Elizabeth Ross**

Australia

**Tanya Rossi**

Canada

**John Rossmeisl**

USA

**Dennis Rubbenstroth**

Germany

**Till Ruemenapf**

Austria

**Thomas Rutkowski**

USA

**Andrew Rycroft**

UK

**Marie-Pierre Ryser-Degiorgis**

Switzerland

**Mikel Sabater**

Spain

**Claude Sabeta**

South Africa

**Farhad Safarpoor Dehkordi**

Iran

**Musa Sahin**

Turkey

**Orhan Sahin**

USA

**Michelle Sait**

Australia

**Lazaros Sakkas**

Greece

**Yoshihiro Sakoda**

Japan

**Fidel San Román**

Spain

**Rodrigo Sanabria**

Argentina

**Javier Sanchez**

Canada

**Arturo Sanchez-Paz**

Mexico

**Berta São Braz**

Portugal

**Rafal Sapierzynski**

Poland

**Manolis Saridomichelakis**

Greece

**Tomohiro Sasanami**

Japan

**Jimmy Saunders**

Belgium

**Ioannis Savvas**

Greece

**Claire Scantlebury**

UK

**Eugenio Scanziani**

Italy

**Sabine Schäfer-Somi**

Austria

**Kathrin Schemann**

Australia

**Julie Schmied**

Canada

**Lauren Schnabel**

USA

**Stephan Schumacher**

Germany

**Gerald Fritz Schusser**

Germany

**Stefan Schwarz**

Germany

**Heinzpeter Schwermer**

Switzerland

**Ignacio Segarra**

Spain

**Alessandro Seguino**

UK

**Hermann Seifert**

Germany

**Tsutomu Sekizaki**

Japan

**Thomas Selhorst**

Germany

**Kim Selting**

USA

**Martin Sergeant**

UK

**Torsten Seuberlich**

Switzerland

**Hans-Martin Seyfert**

Germany

**Nesrin Seyhan**

Turkey

**Tongling Shan**

China

**Sudarvili Shanthalingam**

USA

**Alexandra Shaw**

UK

**Wanyu Shi**

China

**Workineh Shibeshi**

Ethiopia

**Barbara Shock**

USA

**Meng Siak**

Australia

**Marina Sibila**

Spain

**Alexandre Rodrigues Silva**

Brazil

**Pedro Silva**

Brazil

**Rodrigo Otávio Silva**

Brazil

**Hubert Simhofer**

Austria

**Nagendrakumar Singanallur**

Australia

**Mini Singh**

Australia

**Raj Kumar Singh**

India

**Pavel Siroky**

Czech Republic

**Patchima Sithisarn**

Thailand

**Katherine Skorupski**

USA

**Philip Skuce**

UK

**Jan Slapeta**

Australia

**Josh Slater**

UK

**Ronald Sluyter**

Australia

**Hilde Smith**

Netherlands

**Richard Smith**

UK

**Laura Soler Vasco**

Belgium

**Anongnart Somchit-Assavacheep**

Thailand

**Kun-Ho Song**

Korea, South

**Victoria Soroker**

UK

**Eva Spada**

Italy

**Jessica Sparkes**

Australia

**Monica Sparo**

Argentina

**Joachim Spergser**

Austria

**Subramaniam Srikumaran**

USA

**Judith Stack**

UK

**Francesco Staffieri**

Italy

**Jessica Stahl**

Germany

**Karl Stahl**

Sweden

**Catherine Stalin**

UK

**Amy L Stanton**

USA

**Christoph Staubach**

Germany

**Jenny Stavisky**

UK

**Frik Stegmann**

South Africa

**Timothy Stein**

USA

**Sarah Steinbach**

Germany

**Hs Steinberg**

USA

**Jevrosima Stevanovic**

Serbia

**Kim Stevens**

UK

**Mark Stevenson**

Australia

**Mark Stevenson**

New Zealand

**George Stilwell**

Portugal

**Reinhard Sting**

Germany

**Norbert Stockhofe-Zurwieden**

Netherlands

**Diana Stone**

Grenada

**Viola Strompfová**

Slovakia

**Rebecca Strong**

UK

**Shuo Su**

China

**Hiromu Sugiyama**

Japan

**Jianhe Sun**

China

**Lakshmi Sunkara**

USA

**Gila Sutton**

Israel

**Pavol Svorc**

Slovakia

**Emmanuel Swai**

Tanzania

**Kelly Swanson**

USA

**Jane Sykes**

USA

**Monika Szymanska-Czerwinska**

Poland

**Sabine Tacke**

Germany

**John S. Tam**

Hong Kong

**Qinghai Tang**

China

**Florence Tardy**

France

**Mike Taylor**

UK

**Marta Teixeira**

Brazil

**Marisa Tellez**

USA

**Doga Temizsoylu**

Turkey

**Calogero Terregino**

Italy

**Eileen Thacker**

USA

**Douglas Thamm**

USA

**Alexandre Thibodeau**

Canada

**Charles Thoen**

USA

**Peter Thompson**

South Africa

**Peter T. Thomsen**

Denmark

**Gavin Thomson**

Zambia

**Barbara Thur**

Switzerland

**Deena Tiches**

USA

**Andrea Tipold**

Germany

**Laurent Tiret**

France

**Mike Tokach**

USA

**Montserrat Torremorell**

USA

**Sabine Totemeyer**

UK

**Philippe Totte**

France

**Pierre-Louis Toutain**

France

**Imke Traulsen**

Germany

**Paolo Trevisi**

Italy

**Annabelle Troegeler**

France

**Troy Trumble**

USA

**Marianna Tryfonidou**

Netherlands

**Panagiotis Tsikouras**

Greece

**Romana Turk**

Croatia

**Stanislawa Tylewska-Wierzbanowska**

Poland

**Jouni Uitto**

USA

**Reiner Ulrich**

Germany

**Hermann Unger**

Austria

**Melissa Upjohn**

UK

**Yves Van Der Stede**

Belgium

**Leo Van Leengoed**

Netherlands

**Hugo Van Oostrom**

Netherlands

**Paul René Van Weeren**

Netherlands

**Katrien Vanderperren**

Belgium

**Raphael Vanderstichel**

Canada

**Marc Vandevelde**

Switzerland

**Jean-Michel Vandeweerd**

Belgium

**An Vanhaesebrouck**

UK

**Mary Varaschin**

Brazil

**Luigi Varesio**

Italy

**Celina Vega**

Argentina

**Monica Venner**

Germany

**Claudio Vergari**

France

**Tiziano Verri**

Italy

**Haralabos Ververidis**

Greece

**Flavie Vial**

Switzerland

**Joaquin Vicente**

Spain

**Enric Vidal**

Spain

**Carlos Viegas**

Portugal

**Jose Manuel Vilar**

Spain

**Amy Vincent**

USA

**Charles Vite**

USA

**Anastasia Vlasova**

USA

**Andrea Vögtlin**

Switzerland

**Brigitte Von Rechenberg**

Switzerland

**Zoran Vrbanac**

Croatia

**Joe Wakshlag**

USA

**Cheryl Waldner**

Canada

**Per Wallgren**

Sweden

**John Walmsley**

UK

**Michael Wang**

USA

**Hui Wang**

USA

**Jiaqi Wang**

China

**Qin Wang**

China

**Scott Weese**

Canada

**Patrick Wefstaedt**

Germany

**Qiang Wei**

China

**Marleen Werkman**

UK

**Kerstin Wernike**

Germany

**Björn Weström**

Sweden

**Ulrik Westrup**

Denmark

**Nick Wheelhouse**

UK

**Paul Wichgers Schreur**

Netherlands

**Barbara Wieland**

Mongolia

**Paul Wigley**

UK

**Rebecca Wilkes**

USA

**Colin Wilks**

Australia

**Lucas Willems**

Belgium

**Brian Willett**

UK

**Andrew Williams**

Denmark

**David Williams**

Uganda

**Tim Williams**

UK

**Diana Williams**

UK

**Benjamin Willing**

Canada

**Sandra Wilsher**

UK

**Stephen Wilson**

Belgium

**Angela Witzel**

USA

**R. Mark Wooten**

USA

**Deanna Worley**

USA

**Lihua Xiao**

USA

**Ting Xu**

China

**Seiji Yamamoto**

Japan

**Hanchun Yang**

China

**Qian Yang**

China

**Yuanchao Ye**

USA

**Jung-Yong Yeh**

Korea, South

**Kadir Yesilbag**

Turks and Caicos Islands

**Zeki Yilmaz**

Turkey

**Xin Yin**

China

**Han Sang Yoo**

Korea, South

**Yukinori Yoshimura**

Japan

**Megan Young**

Australia

**Muhammad Shahbaz Yousaf**

Pakistan

**Zhongtang Yu**

USA

**Gui-Hong Zhang**

China

**Jin Zhang**

USA

**Jianqiang Zhang**

USA

**Longxian Zhang**

China

**Shuning Zhang**

USA

**Feng-Qi Zhao**

USA

**Junlong Zhao**

China

**Bin Zhou**

China

**En-Min Zhou**

China

**Xing-Quan Zhu**

China

**Alessandro Zotti**

Italy

**Feng-Cai Zou**

China

**Elizabeth Zumbrun**

USA

**Gila Zur**

Israel

